# Network Location-Aware Service Recommendation with Random Walk in Cyber-Physical Systems

**DOI:** 10.3390/s17092059

**Published:** 2017-09-08

**Authors:** Yuyu Yin, Fangzheng Yu, Yueshen Xu, Lifeng Yu, Jinglong Mu

**Affiliations:** 1 School of Computer Science and Technology, Hangzhou Dianzi University, Hangzhou 310019, China; yinyuyu@hdu.edu.cn (Y.Y.); 151050018@hdu.edu.cn (F.Y.); 2 Key Laboratory of Complex Systems Modeling and Simulation of Ministry of Education, Hangzhou 310019, China; 3 School of Software, Xidian University, Xi’an 710071, China; 4 Hithink RoyalFlush Information Network Co., Ltd., Hangzhou 310023, China; yulifeng@myhexin.com; 5 Fushun Power Supply Branch, State Grid Liaoning Electric Power Supply Co., Ltd., Fushun 113008, China; yuncloud888@gmail.com

**Keywords:** cyber-physical systems, service recommendation, QoS prediction, network location, random walk

## Abstract

Cyber-physical systems (CPS) have received much attention from both academia and industry. An increasing number of functions in CPS are provided in the way of services, which gives rise to an urgent task, that is, how to recommend the suitable services in a huge number of available services in CPS. In traditional service recommendation, collaborative filtering (CF) has been studied in academia, and used in industry. However, there exist several defects that limit the application of CF-based methods in CPS. One is that under the case of high data sparsity, CF-based methods are likely to generate inaccurate prediction results. In this paper, we discover that mining the potential similarity relations among users or services in CPS is really helpful to improve the prediction accuracy. Besides, most of traditional CF-based methods are only capable of using the service invocation records, but ignore the context information, such as network location, which is a typical context in CPS. In this paper, we propose a novel service recommendation method for CPS, which utilizes network location as context information and contains three prediction models using random walking. We conduct sufficient experiments on two real-world datasets, and the results demonstrate the effectiveness of our proposed methods and verify that the network location is indeed useful in QoS prediction.

## 1. Introduction

Cyber-physical systems (CPS) are fitting into modern society. CPS integrate multiple techniques, including distributed computing, communication and automatic control, to support variety of intelligent services and applications in many fields, such as transportation, healthcare, entertainment and city infrastructure [1,2,3]. In CPS, sensors and mobile devices collect various information from the physical environment, and thus generate a huge amount of data. Gartner foresees that there will be 20.4 billion sensors or devices that are connected worldwide in 2020 [4]. Based on the data collected, enterprises can provide services which will improve our life quality. However, due to the huge number of services in CPS, it becomes an urgent task to effectively select the suitable candidate services for different users. Besides the difference of functionality, services in CPSs are likely to provide different quality under various environments, especially in the different network conditions, such as 4G, 5G, and Wi-Fi. In this paper, our goal is to select the suitable services with high quality for users in CPS.

Service-oriented computing (SOC), as a popular distributed computing paradigm, is widely studied by a series of institutions and standardization organizations. Traditionally, the SOC community focuses on service composition [5], service discovery [6], service selection [7,8,9], and service testing [10]. The existing researches are indeed verified to be valuable in many fields, such as distributed computing, cloud computing and cyber-physical system. However, along with the explosion of resources in CPS, it becomes increasingly difficult for a user to find the suitable services. In the case of a huge data volume, it is urgent to develop an effective service recommender system. In this paper, we focus on designing an applicable service recommendation method for CPS.

There are two types of properties for a service, functional properties and non-functional properties. The non-functional properties are represented by quality-of-service (QoS), such as response time, throughput and reliability [11]. In recent years, study on QoS is an important topic in service computing and distributed computing. Since a service usually runs in a dynamic network, the QoS value of a service is likely to change due to the instability of the network. Thus, it is important to predict QoS values for achieving effective service recommendation. In this paper, we use the historical invocation records of users and services to predict QoS values. We can build a user-service invocation matrix (a toy example is shown in Table 1), in which each entry represents the response time value. The entries with the question mark “?” indicate that the target QoS values are unknown. Compared to the large number of services, the number of services that are invoked by a user is rather small, which leads to the high sparsity of the user-service matrix.

Collaborative filtering (CF) is a prediction algorithm that is employed in QoS prediction [12,13,14]. The CF algorithm utilizes the user consumption records to identify similar neighbors, and predicts the preference of the target user using the preferences of similar neighbors. In service recommendation, the CF algorithm utilizes the invocation records of the target user to identify similar neighbors, and further to predict the QoS values. The existing research has found that the traditional CF algorithms are hard to generate results with high accuracy, under the case that the invocation records are sparse, which is the normal case in CPS [1]. However, there are few works that try to solve this problem. Meanwhile, the QoS of a service is heavily impacted by the network location. It can be inferred that if two services or two users have the similar network location, the QoS values generated from the same invocations are likely to be similar. To solve the service recommendation problem, in this paper, we make the following contributions:

1. We propose three novel prediction models, which are the user-based random walk model, service-based random walk model and a hybrid model. All of the proposed models have the capability of utilizing the network location information in CPS. Also, our proposed models can find the user groups or service groups in which members share potential similarity.
2. We propose an extended similarity computation method based on Euclidean distance, which is verified to be effective in solving the ‘cold-start’ problem.
3. We propose a similar neighbor selection algorithm, which integrates the network location, to filter the fake neighbors with abnormal QoS values.
4. We conduct sufficient experiments on real-world datasets, and the experimental results demonstrate the effectiveness of our proposed models.

The rest of this paper is organized as follows. Section 2 summarizes the related work. Section 3 presents our motivation. Section 4 introduces the base model and technique used in this paper. Section 5 elaborates the proposed prediction models and similarity computation method. Section 6 reports the experimental results and gives analysis. Section 7 concludes the paper and discusses the future work.

## 2. Related Work

Collaborative filtering (CF) is widely used in service recommendation. Shao et al. [15] standardized the QoS value and proposed a user-specific CF algorithm. Chen et al. [16] proposed a hybrid model, which integrated service-based CF and latent semantic analysis. Jiang et al. [17] also proposed a CF-based hybrid model, which was a linear combination of user-based CF and service-based CF. The authors also extended the traditional PCC (Pearson Correlation Coefficient).

Some researchers have found that the context information, such as network location and geographical location, is closely related to the QoS of a service [18]. Some researchers integrated such context information into the CF algorithm, achieving better prediction accuracy. Chen et al. [19] used the bottom-up hierarchical clustering algorithm with utilizing the user geographical location to mine the similar region, and further integrated the region information into the CF algorithm. Liu et al. [20] proposed a location-aware CF algorithm, which identified each potential autonomous system (AS) based on the IP addresses of users and services. The proposed algorithm partitioned the users and services into different groups according to the corresponding autonomous system, to select similar neighbors. The authors also extended the PCC and the CF algorithm, to reflect the similarity relationship in the same autonomous system. Yu et al. [21] proposed an extended latent semantic analysis model utilizing network location for service recommendation. The latent semantic analysis model is also widely used in recommender systems, and has high flexibility and diversity. In detail, the authors modeled the network location information to the regularization term, and factorized the proposed model as SVD (Singular Value Decomposition). Yao et al. [22] proposed a content-based CF algorithm, which utilized the description content extracted from the WSDL (Web Services Description Language) files, to mine the user preference for service invocation. He et al. [23] designed a location-based hierarchical matrix factorization (HMF) method for service recommendation to acquire high accuracy QoS prediction. In addition, the time factor plays a very important role in QoS prediction. Yu et al. [24] proposed a time-aware CF for QoS-based service recommendation algorithm to calculate the missing QoS values. In order to handle cold start problem in service recommendation, Lee et al. proposed a location-based matrix factorization technique via preference propagation (LMF-PP) [25]. LMF-PP used the local information and preference propagation to get exact QoS prediction. Qi et al. [26] proposed a novel matrix factorization approach which integrated both network and service neighborhood information into matrix factorization. Ma et al. proposed a QoS prediction approach for multi-dimensional QoS data which considered all the dimensions of QoS data and performed the prediction [27]. The authors in [28] proposed a credibility-aware QoS prediction method (named CAP) to solve the problem. CAP used two-phase K-means clustering to identify the untrustworthy QoS values, then predicted the missing QoS values by the credible clustering information. Zhou et al. proposed a CF model, which was based on the PGraph to represent neighborhood [29].

Note that, under the case of high data sparsity, CF and its extended algorithms are likely to achieve quite low prediction accuracy. In recent years, some researchers also try to solve the recommendation deficiency issue in high sparsity case. Zhang et al. [30] extended the CF algorithm by designing a sparse matrix completion method. The proposed algorithm first observed the missing value, and filled a value according to the predefined rules in an iterative manner. Yildirim and Krishnamoorthy [31] proposed a random walk-based movie recommendation model. In the model proposed the authors first computed the similarity between each pair of items, then took each item as the nodes, and the similarity as the weight on the edge, to build an undirect graph, and modeled the computation process as a Markov process. Although the model proposed by the authors can partly alleviate the sparsity issue, this model is not capable of using the context information. Hu et al. [32] proposed a novel time-aware approach, which took time information into account to measure the similarity between users and services respectively. Xie et al. [33] proposed an asymmetric correlation regularized matrix factorization (MF) to alleviate the data sparsity problem. In contrast, our proposed models are designed to utilize the network location as the context information, and extend the random walk model, both to solve the sparsity issue.

## 3. Research Motivation

### 3.1. The Sparsity Issue

**Figure 1:**
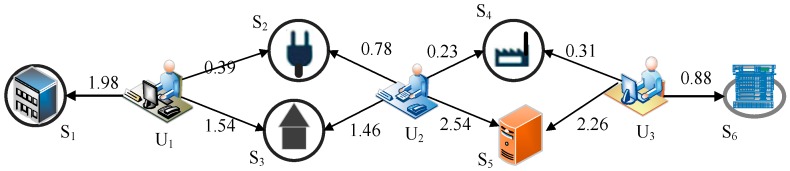
The real-world service invocation scenario in cyber-physical systems (CPS).

In CPS, due to the large number of services and limited invocation experiences, the number of services that are commonly invoked by users is usually quite small. The real-world service invocation scenario is shown in Figure 1.

In Figure 1, there are three users and six services in CPS, and here we take the response time as the QoS property to explain. User 1 and user 2 both invoke service 2 and service 3, and user 2 and user 3 both invoke service 4 and service 5. User 1 and user 3 do not have any service both invoked. If we adopt the PCC to compute the similarity of users, and similarity result of user 1 and user 2 is close to 1, and the similarity result of user 2 and user 3 is also close to 1. It can be inferred that user 1 and user 2 are similar to each other, and user 2 and user 3 are also similar. But note that, since user 1 and user 3 do not share any invocation experience, the similarity of user 1 and user 3, which is directly computed according to the QoS records, is 0. However, due to the fact that user 1 and user 2 are similar, and user 2 and user 3 are also similar, it can be inferred that user 1 and user 3 are also similar. In this paper, we call the similarity that is computed directly from the known QoS values the direct similarity. In many cases, it is not enough to reflect the real similarity only using direct similarity.

In this paper, our idea is to employ the transitive relation computation to acquire the real similarity. We use graph as the basic data structure to model the transitive relation. In the graph, a user is a node, and if the direct similarity of two users is not 0, there will be an edge connecting the two nodes in the graph. We employ the random walk model to find the potential similarity among nodes without direct connection. In detail, we use the converged probability after the random walk process to represent the similarity of two nodes, which is regarded as the real similarity. Besides, since the network location is a key factor that influences the QoS value, we integrate the network location into the state transition process of random walk model. Further, we propose two network location-based random walk models, specific to the user side and service side, respectively.

### 3.2. Similarity Computation

In ordinary service recommendation methods, Pearson correlation coefficient (PCC) is commonly used to compute similarity. PCC reflects the linear correlation of the rating records of different users, and such correlation reflects the similarity of the preferences, and the assumption is that the user preference will be unchanged for a long time. In contrast, in service recommendation, since the network environment usually changes dynamically. For example, the connection speed and network stability are likely to change, which leads to the change of QoS. So it is unsuitable to use linear correlation to measure the user similarity, and PCC is not used in this paper as the similarity measure. The Euclidean distance measures the difference of the multi-dimensional data, including both linear and non-linear correlation. In this paper, we extend the Euclidean distance by introducing a penalty factor, and further propose a user-based similarity measure and a service-based similarity measure.

### 3.3. Network Location-Based Neighbor Selection

In CF-based models, the prediction for QoS relies on similar neighbor selection, so the neighbor selection technique largely impacts the recommendation result. In the same autonomous system, the users are likely to receive similar QoS values during the same invocation process. Here is an example. The response time is largely decided by the network bandwidth and network location. In the same autonomous system, users or services usually share the same network condition, and thus tend to experience similar QoS. In this paper, for similar neighbor selection, we fully employ the network location information, such as the autonomous system and country affiliation.

## 4. Base Model and Technique

### 4.1. Collaborative Filtering

Collaborative filtering (CF) is a widely used prediction algorithm in QoS prediction. The CF algorithm mines a user’s preference based on the preferences of similar neighbors, and predicts the ratings based on the mined preference. Let $U=\left\{u_{1},u_{2},\ldots ,u_{m}\right\}$ be the user set, and $I=\left\{i_{1},i_{2},\ldots ,i_{n}\right\}$ be the item set, where *m* and *n* are the number of users and services, respectively.

The CF algorithm can be classified into two categories, that is, the neighborhood-based CF algorithm (also named the memory-based CF algorithm) and the model-based CF algorithm. The idea of a neighborhood-based CF algorithm is to identify a similar neighborhood for the target user or service, and predict the missing ratings using the rating records of the neighborhood. The neighborhood-based CF algorithm can be further classified into two fine-grained categories, that is, the user-based CF algorithm and the item-based CF algorithm.

### 4.2. Network Location

**Figure 2:**
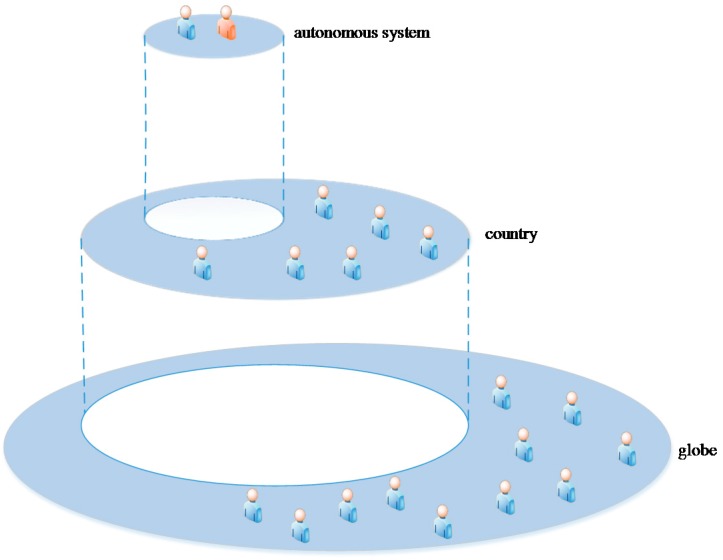
Network location-based user partition in CPS.

In CPS, the QoS of a service is quite related to the network environment. If we take an autonomous system (AS) as the unit, the users and services in the same autonomous system will adopt the same routing protocol. That is, an autonomous system can be regarded as a network environment consisting of the routers that are controlled by the same provider. The network configuration in the same autonomous system, such as network bandwidth and network stability, is shared by all users and services, so the QoS values received by users are likely to be similar. We give an autonomous system in the following Figure 2.

### 4.3. Random Walk

Random walk is a probabilistic model integrating graph theory and stochastic process, which is used to analyze the transitivity of relations [34]. Random walk can be depicted using the Markov chain model, which depicts the random process of discrete data. Markov chain can be modeled as a state sequence, in which each state is decided by several limited previous states. In random walk, a directed graph is constructed to represent a state space, and each node represents a state. There is a transition probability between each pair of nodes, and all transition probabilities form the transition probability matrix. Starting from one node, the state sequence is generated according to the transition probability matrix along with the random walk process $\left(v_{t}:t=0,1,\ldots \right)$. Usually, the random walk process follows the first-order Markov chain, that is, the probability that the current state *t* is in node *i* is only decided by the node of the state.

## 5. The Proposed Prediction Models

### 5.1. The QoS Prediction Framework

**Figure 3:**
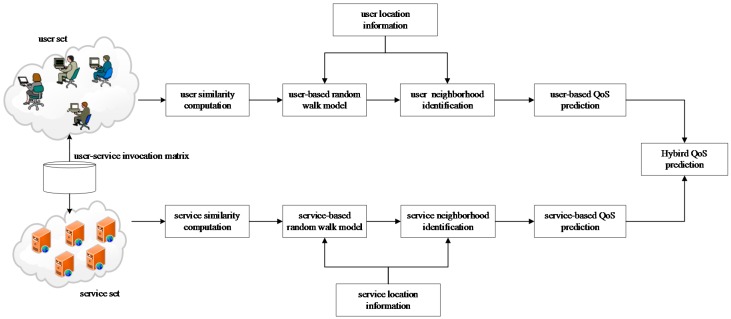
The proposed quality-of-service (QoS) prediction framework.

To recommend services with high quality, our paper focuses on achieving QoS prediction with high accuracy. Here is the proposed framework of QoS prediction (Figure 3). The proposed QoS prediction models include the user-based prediction model, the service-based prediction model and a hybrid model, which are stated below.

1. **The user-based prediction model**. This model extends the user-based CF model, which improves the user similarity computation by integrating random walk model, to select similar neighbors. Both the random walk model and neighbor selection are capable of using the network location information. The unknown QoS values are predicted using the QoS records of similar neighbors collaboratively.
2. **The service-based prediction model**. This model extends the service-based CF model, which improves the service similarity computation also by integrating random walk model. Other technical details are similar to those of the user-based prediction model.
3. **The hybrid model**. To further improve the prediction accuracy, our idea is to fully utilize the similar user neighborhood and similar service neighborhood. In this paper, we propose a linear hybrid model, which combines the predicted results of the user-based model and service-based model.

### 5.2. Direct Similarity Computation

The Euclidean distance is a multi-dimensional distance computation method, and can be easily extended to measure the distance of QoS values.

(1) $$\bar{distance}=\frac{1}{\left|S_{uv}\right|}\sqrt{\sum_{i\in S_{uv}}{\left(q_{ui}-q_{vi}\right)}^{2}}$$

where $S_{uv}$ represents the service set, in which the services are commonly invoked by user *u* and user *v*. A smaller value of Equation (1) means higher similarity. So the similarity of user *u* and user *v* can be computed as the reciprocal of the Euclidean distance of the invocation records, which is shown as follows.

(2) $$sim(u,v)=\frac{1}{1+\frac{1}{\left|S_{uv}\right|}\sqrt{\sum_{i\in S_{uv}}{\left(q_{ui}-q_{vi}\right)}^{2}}}$$

where the number 1 in the denominator is a way of Laplacian smooth to avoid the denominator being 0. $q_{ui}$, $q_{vi}$ are the QoS values of user *u* to service *i* and user *v* to service *i*, respectively. It can be seen that the high closeness of the invocation records will lead to high similarity. However, Equation (2) has two defects:

1. The fluctuation range of QoS values can be very large. For example, the response time value may be any value in the range of 0 s~20 s or 30 s. If two users receive very different QoS values from the same service, the final similarity can be generated with a large bias.
2. In the case that the number of services commonly invoked by two users is small, the similarity computation result tends to be vulnerable. In an extreme case that two users only share one service that is commonly invoked, the similarity result turns to be quite unreliable.

To solve the above issues, we improve Equation (2) as follows.

(3) $$sim(u,v)=\frac{1}{1+\frac{1}{\left|S_{uv}\right|}\sqrt{\sum_{i\in S_{uv}}{\left(\left(q_{ui}-{\bar{q}}_{i}\right)-\left(q_{vi}-{\bar{q}}_{i}\right)\right)}^{2}}}$$

where ${\bar{q}}_{i}$ is the average value of QoS values received by user *u* and user *v* invoking service *i*. Similar to the user similarity computation, the service similarity is computed as

(4) $$sim(i,j)=\frac{1}{1+\frac{1}{\left|U_{ij}\right|}\sqrt{\sum_{u\in U_{ij}}{\left(\left(r_{ui}-{\bar{q}}_{u}\right)-\left(r_{uj}-{\bar{q}}_{u}\right)\right)}^{2}}}$$

where $r_{ui}$ and $r_{uj}$ are the QoS values of user *u* invoking service *i* and service *j* respectively. ${\bar{q}}_{u}$ denotes the average value of the QoS values of user *u* invoking services *i* and *j*.

### 5.3. The Proposed Random Walk Models

**Figure 4:**
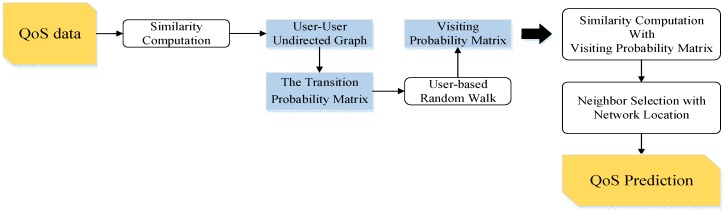
The procedure of user-based random walk model.

Although we improve the similarity computation in Section 5.2, Equations (3) and (4) focus on the direct similarity computation (i.e., only using the QoS records), and are not capable of reflecting the real similarity. To solve this problem, our paper builds user-based random walk model and service-based random walk model. Our idea is to use the state transition on the undirected graph to represent the transition of similarity relationship. We take the user-based random walk model as the example to give the detailed explanation, and the computation procedure is shown in the following Figure 4.

First, using Equation (3), we have computed the similarity of users and have built the user-to-user undirected graph. Then, based on the similarity relation and network location, we compute the transition probability to form the transition probability matrix. Further we propose the user-based random walk model, taking the transition probability matrix as the input to compute the visiting probability matrix, which is used to improve the similarity computation of users. In the end, with the computed similarity and network location information, we select the final neighbors and complete the QoS prediction.

#### 5.3.1. The State Transition of Random Walk

**Figure 5:**
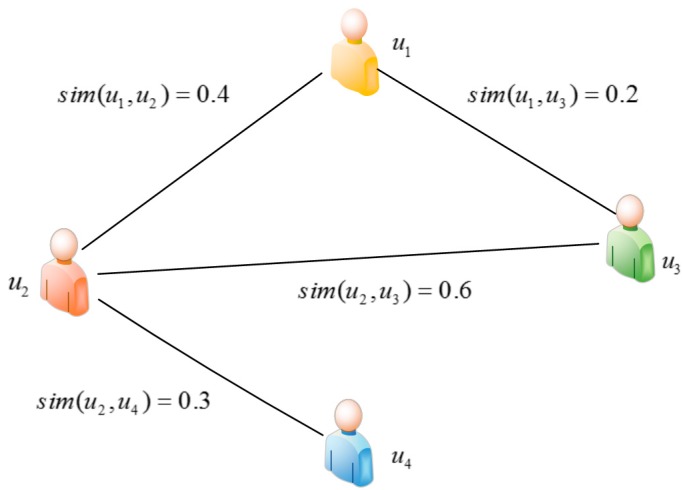
The undirected graph of user similarity.

In the graph, a user is a node, and the direct similarity of two users is the weight of the edge. Based on that, we build the undirected graph $G\left(V_{u},E\right)$ and use Markov chain to model the state transition of random walk. A toy example of the undirected graph is given in Figure 5. Let $u_{0}\in V_{u}$ be the start node, and the initial state be $X_{0}=u_{0}$, representing that the initial state of random walk is in node $u_{0}$. The state transition process after *k* steps is $X_{0}\to X_{1}\to \ldots \to X_{k-1}\to X_{k}$. The state of the *k*th step is only decided by the state of the *k* − 1th step. Assume that the node, which the *k* − 1th step is in, is user u, and the node that the *k*th step is in is user v. The transition probability from node u to node v is

(5) $$prob(u,v)=Pr(X_{k}=v|X_{k-1}=u)$$

Note that, the weight of the edge connecting node u and node v is computed by Equation (3), and following that, we define the transition probability from node u to node v as

(6) $$prob(u,v)=Pr(X_{k}=v|X_{k-1}=u)=\frac{sim(u,v)}{\sum_{v^{\prime }\in adj\left(u\right)}sim(u,v^{\prime })}$$

where adj(u) is the adjacent node set of user u, sim(u,v) is the similarity of user u and user v, and sim(u,v) is the similarity of user u and the adjacent user $v_{0}$. A higher similarity means a larger transition probability.

Since the network configurations of users that are in the same autonomous system are likely to be similar, we aim to increase the transition probability among these users to enhance the similarity computation. In detail, we propose a transition weight ω by introducing the network location information. For two adjacent users u and v, the corresponding weight ω is computed with

(7) $$\omega \left(u,v\right)=\frac{sim\left(u,U\right)+sim\left(v,U\right)}{2}$$

where sim(u,U) represents the similarity of QoS values of user *u* and the average QoS values in set *U*. The set *U* contains a certain number of users, and is decided by the following rules.

1. If user u and user v are in the same autonomous system, then the set *U* contains the users of the autonomous system.
2. If user u and user v are in the same country, but not the same autonomous system, then the set *U* contains the users of the country.
3. If user u and user v are not in the same country or autonomous system, then the set *U* contains all users.

After the weight computation, the transition probability in Equation (6) is enhanced to

(8) $$prob(u,v)=\frac{\omega (u,v)\times sim(u,v)}{\sum_{v^{\prime }\in adj\left(u\right)}\omega \left(u,v^{\prime }\right)\times sim(u,v^{\prime })}$$

Based on the transition probability of the adjacent users, we construct a transition probability matrix $M\left(M\in {\mathbb{R}}^{m\times m}\right)$.

$$M=\left[\begin{array}{ccc} M_{11} & \ldots & M_{1m}\\ \vdots & \ddots & \vdots \\ M_{m1} & \ldots & M_{mm} \end{array}\right]$$

where each entry $M_{uv}$ (1≤u,v≤m) represents the transition probability prob(u,v). We give a detailed example based on Figure 4. There are four users in Figure 4, and we compute the similarity between each pair of users, and construct the transition probability matrix as follows.

$$\left[\begin{array}{cccc} 0 & \frac{2}{3} & \frac{1}{3} & 0\\ \frac{4}{13} & 0 & \frac{6}{13} & \frac{3}{13}\\ \frac{1}{4} & \frac{3}{4} & 0 & 0\\ 0 & 1 & 0 & 0 \end{array}\right]$$

#### 5.3.2. The Transition Probability Matrix

Let $Pr\left(X_{k}=v|X_{0}=u_{0}\right)$ represent the conditional probability of starting from node $u_{0}$ to reach node v after k steps. Following the Bayesian rule, we can get

(9) $$Pr(X_{k}=v|X_{0}=u_{0})=\sum_{v^{\prime }\in V_{u}}Pr(X_{k-1}=v^{\prime }|X_{0}=u_{0})Pr(X_{k}=v|X_{k-1}=v^{\prime })$$

There are two choices at each node during random walking:

1. Continue the walking process along a route with the probability α.
2. Skip back to the initial node $u_{0}$ with probability 1−α. In this paper, α is assigned to 0.85 following the suggestion of Page et al. [35].

In detail, if node v is not node $u_{0}$ ($v\neq u_{0}$, the first case), then we have

(10) $$\begin{array}{ll} Pr(X_{k}=v|X_{0}=u_{0}) & =\sum_{v^{\prime }?V_{u}}Pr(X_{k-1}=v^{\prime }|X_{0}=u_{0})Pr\left(X_{k}=v|X_{k-1}=v^{\prime }\right)\\ & =a^{k}M^{k-1}P_{0}M_{.v}^{T} \end{array}$$

If node v is node $u_{0}$ ($v=u_{0}$, the second case), then we have

(11) $$Pr(X_{k}=v|X_{0}=u_{0})=\alpha^{k}M^{k-1}P_{0}M_{.v}^{T}+\left(1-\alpha \right)$$

In summary, we have

(12) $$Pr(X_{k}=v|X_{0}=u_{0})=\left\{ \begin{array}{l} \alpha^{k}M^{k-1}P_{0}M_{.v}^{T},v\neq u_{0}\\ \alpha^{k}M^{k-1}P_{0}M_{.v}^{T}+\left(1-\alpha \right),v=u_{0} \end{array}\right.$$

where $M_{.v}^{T}$ is the vth column vector of the probability transition matrix M, and each element in $M_{.v}$ represents the transition probability of a certain node moving to node v. $P_{0}$ is the initial probability distribution vector in random walk. In the beginning of random walk, there is only one node as the start node, that is, the probability starting from the current node is 1, and from other nodes is 0. Take the toy example in Figure 5 to explain. Assume that the target node is node $u_{1}$, then the initial probability distribution vector is

$$P_{0}=\left(1,0,0,0\right)$$

In another case, if the target node is node $u_{2}$, then the initial vector is

$$P_{0}=\left(0,1,0,0\right)$$

The rest of cases that the target node is some other nodes can be done in the same way.

Considering that it will lead to high complexity if we use Equation (12) directly, in this paper, we construct a matrix to organize the whole probability distribution. At the beginning of the random walk, only one node is selected to be the start node, then the initial probability distribution matrix is

$$P_{0}=\left[\begin{array}{ccc} 1 & \ldots & 0\\ \vdots & \ddots & \vdots \\ 0 & \cdots & 1 \end{array}\right]$$

If the number of users is M, and the initial probability distribution matrix $P_{0}$ is identity matrix $\left(P_{0}\in R^{m\times m}\right)$. Based on Equation (12), the probability distribution matrix after random walking with k steps is

(13) $$P_{k}=(1-\alpha )P_{0}+\alpha M^{T}P_{k-1}$$

Along with the step k being infinite, the probability will converge to be stable, which is decided by the steady state distribution of the Markov chain. Equation (13) can be further transformed into the following Equation (14), and the visiting probability matrix $P^{*}$.

(14) $$P^{*}=(1-\alpha ){\left(I-\alpha M^{T}\right)}^{-1}P_{0}$$

The vising probability reflects the improved user similarity. A larger visiting probability means a higher similarity.

#### 5.3.3. Similarity Computation with Visiting Probability Matrix

Based on the observation that the visiting probability is positively correlated to the similarity, we propose an optimized similarity computation method. First, we compute the neighborhood weight of a user.

(15) $$\varphi_{u}=\frac{1}{\left|adj(u)\right|}\sum_{u^{\prime }\in adj\left(u\right)}\frac{sim(u,u^{\prime })}{P_{uu^{\prime }}^{*}}$$

(16) $$\varphi_{v}=\frac{1}{\left|adj(v)\right|}\sum_{v^{\prime }\in adj\left(v\right)}\frac{sim(v,v^{\prime })}{P_{vv^{\prime }}^{*}}$$

where in Equation (15), adj(u) represents the set of adjacent nodes of user u, and $P_{uu^{\prime }}^{*}$ is the visiting probability of user u to $u^{\prime }$, and $\varphi_{u}$ represents the average ratio of the direct similarity to the visiting probability along the walking route of u. Remember that the user direct similarity is computed by Equation (3) (see Section 5.2). The symbols in Equation (16) have similar meanings. The final similarity of user u and v is computed with

(17) $${sim}^{*}\left(u,v\right)=\frac{\varphi_{u}\times P_{uv}^{*}+\varphi_{v}\times P_{vu}^{*}}{2}$$

where $P_{uv}^{*}$ represents the probability of user u being the start node and visiting user v. $P_{uv}^{*}$ represents the probability of user v being the start node and visiting user u. Using Equation (17), we can compute the similarity of two users that have no common invoked services.

Note that, similar to the user-based random walk model, the service-based random walk model also uses the undirect graph to enhance the service similarity computation. The computation procedure is the same as that explained in this section.

### 5.4. Neighborhood Construction and QoS Prediction

#### 5.4.1. Neighbor Selection with Network Location

It is an important step to select similar neighbors for neighborhood-based CF. The number of neighbors and selection method directly affect the prediction accuracy. There are two methods that are widely used for neighbor selection, topK method and threshold method. The topK method selects *K* users that are most similar to the target user, as his or her neighbors. The threshold method predefines a threshold, and a user whose similarity is higher than the threshold will be selected to be a neighbor of the target user. However, the traditional neighbor selection methods usually only utilize the service invocation records, but fail to utilize the context information.

To solve this issue, this paper integrates the network location with the topK method. In the proposed method, let A(u) be the subset of users who are in the same autonomous system as the target user u. Let U(u) be the subset of all users. The similar neighbor set N(u) of the target user u is generated following the steps below.

Step 1: this step computes the similarity between the target user u and any other user v∈A(u), and then find the *K* most similar users in A(u). The current neighbors in step 1 form the neighbor set NA(u).

Step 2: Besides the neighbors in NA(u) that are selected in step 1, we also select the rest neighbors from U(u). We compute the similarity between the target user u and any other user v(v∈U(u)∧v∉A(u)).

#### 5.4.2. QoS Prediction and Service Recommendation

##### User-Based QoS Prediction

Now we have finished two tasks: similarity computation using user-based random walk model and similar neighbor selection N(u). Similar to the CF algorithm, the QoS value $q_{ui}$ of user u invoking service i is predicted by

(18) $$q_{ui}\approx \frac{\sum_{v\in N\left(u\right)}sim^{*}\left(u,v\right)\times q_{vi}}{\sum_{v\in N\left(u\right)}sim^{*}\left(u,v\right)}$$

We name this method as UL-RW (User Location-based Random Walk).

##### Service-Based QoS Prediction

Similar to the user-based prediction, in service-based QoS prediction we can also finish the similarity computation with service-based random walk model, and similar neighbor selection for target service i. Let *N*(*i*)denote the neighbor set of target service *i*. Also based on the CF algorithm, the QoS value is predicted by

(19) $$q_{ui}\approx \frac{\sum_{j\in N\left(i\right)}sim^{*}\left(i,j\right)\times q_{uj}}{\sum_{j\in N\left(i\right)}sim^{*}\left(i,j\right)}$$

We name this method as SL-RW (Service Location-based Random Walk).

##### Hybrid QoS Prediction

To fully take advantage of the context information on both the user side and service side, we further propose a hybrid model, which combines the results of the UL-RW model and the SL-RW model. We name the model as HL-RW (Hybrid Location-based Random Walk), which is shown below.

(20) $$q_{ui}=\lambda \times q_{ui}^{UL-RW}+(1-\lambda )\times q_{ui}^{SL-RW}$$

where $q_{ui}^{UL-RW}$ and $q_{ui}^{SL-RW}$ are the prediction values of UL-RW model and SL-RW model, respectively. The parameter *λ* is used to balance the weight of the two models in the final result. The sensitivity of our model to *λ* will be studied in the experiment section.

## 6. Experiment and Evaluation

We conduct a series of experiments on two large-scale real-world QoS datasets, to evaluate the prediction accuracy of the proposed models, as well as compared to several well-known methods. We aim to address the following questions.

1. How do the proposed models behave in different data sparsity cases?
2. How do the proposed models perform compared to other models?
3. How do the parameter *λ* and *K* impact the prediction accuracy?

### 6.1. Dataset

In our experiments, we use two real-world service datasets that are published by Zheng et al. [36]. One is the response time dataset, and the other is the throughput dataset. In the two datasets, there are 339 users, 5825 services, and 1,974,675 QoS records. The dataset also contains the geographical location information and network location information, in both the user side and service side. The geographical location information contains a user’s longitude and latitude. The network information contains the IP address of each user and the WSDL file address of each service. The data statistics of this dataset are shown in the following Table 2.

Note that, this dataset does not contain the autonomous system (AS) information of users or services. To supplement these data, we take a user’s IP address and a service’s WSDL file as the input to a public database GeoLite, to look up the corresponding AS number. In this way, we get 137 AS numbers for users and 1021 AS numbers for services.

To evaluate the prediction performance of our models under different training set densities, we randomly select a part of invocation records from the whole dataset. We generate four training sets with different densities, including 5%, 10%, 15% and 20%. Take the 10% case as the example. We randomly extract 10% QoS records from the whole dataset as the training set. The rest of data form the testing set. To make the experimental results more reliable, we repeat the generation of training and testing sets for 100 times, and report the average experimental results in this section.

### 6.2. Evaluation Metric and Parameter Setting

We use MAE (Mean Absolute Error) and NMAE (Normalized Mean Absolute Error) to measure the prediction accuracy of our proposed models. MAE is defined as

$$\mathrm{MAE}=\frac{1}{N}\sum_{u,i}|q_{ui}-{\hat{q}}_{ui}|$$

where $q_{ui}$ represents the real QoS value, ${\hat{q}}_{ui}$ represents the prediction result, and *N* is the number of values in the testing set. A smaller MAE value means higher prediction accuracy. NMAE is computed as the MAE normalized by the mean of all values, which is defined as

$$\mathrm{NMAE}=\frac{\mathrm{MAE}}{\sum_{u,i}q_{ui}/N}$$

For parameter setting, we set the neighborhood size *K* in UL-RW model and SL-RW model to be 5, and set *λ* in HL-RW model to be 0.6. We will investigate the sensitivity of our models to the parameters in the following sections.

### 6.3. Prediction Accuracy Comparison

We compare our models with several well-known existing models, including

1. **UserMean**: this method uses the average value of each user as the prediction value.
2. **ItemMean**: this method uses the average value of each service as the prediction value.
3. **UPCC** (user-based PCC) [37]: the user-based collaborative filtering algorithm using Pearson correlation coefficient (Resnick et al., 1994).
4. **IPCC** (item-based PCC) [38]: the item-based collaborative filtering algorithm using Pearson correlation coefficient (Sarwar et al., 2001).
5. **WSRec** [36]: This method linearly combines the results of UPCC and IPCC to produce a hybrid result (Zheng et al., 2009).
6. **LACF** [39]: A collaborative filtering algorithm that integrates the location information of users and services (Tang et al., 2012).
7. **SVD** [40]: the singular value decomposition model (Koren et al., 2009).

The experimental results are shown in Table 3 (response time dataset) and Table 4 (throughput dataset). From Table 3 and Table 4, we have the following observations.

1. The proposed models SL-RW, UL-RW and HL-RW all achieve smaller MAE and NMAE than the baseline models, almost in all cases of training set densities.
2. Along with the increase in training set density, the prediction accuracy also becomes higher. The reason is that more training data can provide more invocation records to improve the prediction of similarity computation and neighbor selection.
3. The improvement achieved by our models are significant based on the paired *t*-test (*p* < 0.001).

In the rest of this section, we will study the sensitivity of our model to the parameters.

### 6.4. Impact of λ

**Figure 6:**
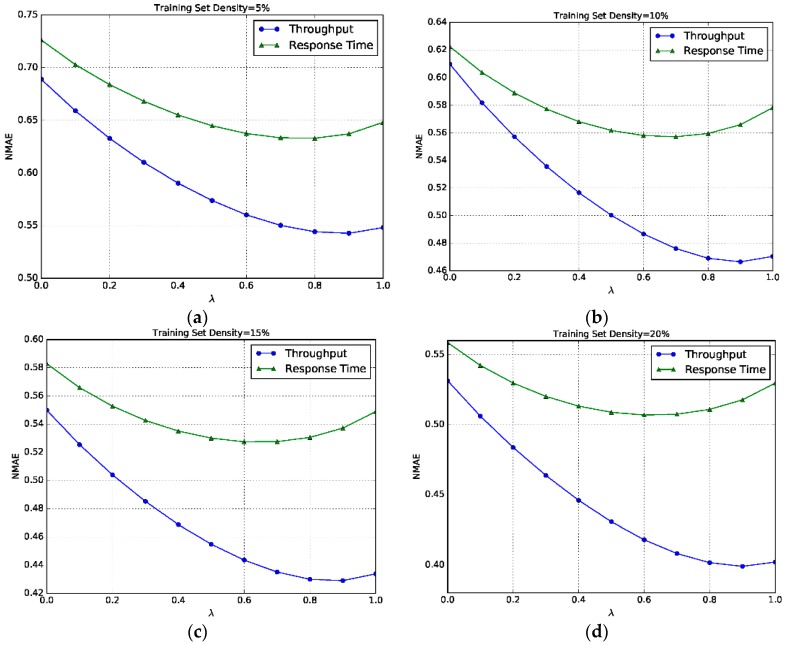
Sensitivity to *λ*. (**a**) Training set density = 5%; (**b**) Training set density = 10%; (**c**) Training set density = 5%; (**d**) Training set density = 10%.

In this section, we investigate the impact of parameter *λ* on the prediction accuracy of model HL-RW. The parameter *λ* controls the weight of UL-RW and SL-RW in the final result. If *λ* is set to 0, the HL-RW model will be degraded to the SL-RW model. In contrast, if *λ* is set to 1, the HL-RW model will be degraded to the UL-RW model. We study the impact of *λ* in the value range of 0 to 1, in both datasets. The other parameter *K* is set to the default value, and the results are shown in the following Figure 6.

Figure 6 shows that for throughput data, the NMAE values first become smaller, and then reach an optimal point at *λ* = 0.9 in most density cases. We can also see that the NMAE value of response time data reaches the optimal point at *λ* = 0.6 in most cases. Recalling Equation (20), it indicates that in the hybrid model, the UL-RW model takes a more important role than the SL-RW model.

### 6.5. Impact of K

**Figure 7:**
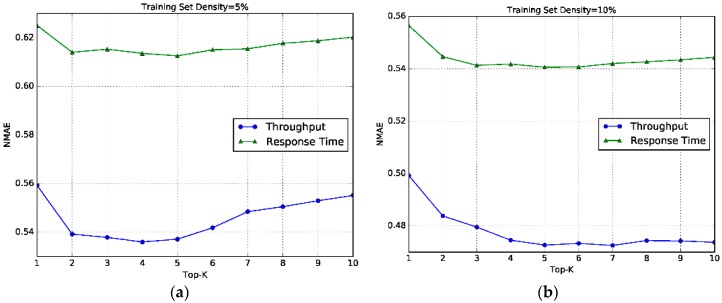

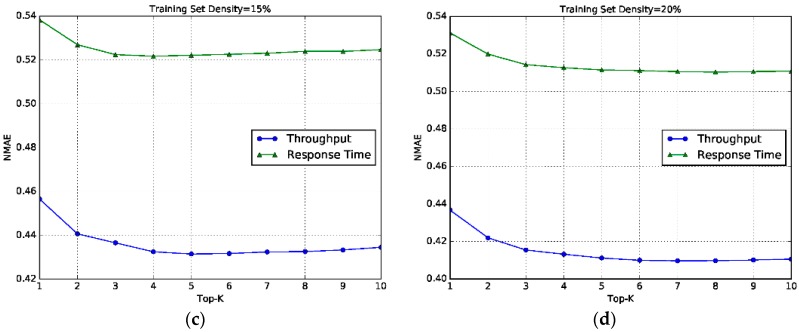
Sensitivity to *K.* (**a**) Training set density = 5%; (**b**) Training set density = 10%; (**c**) Training set density = 15%; (**d**) Training set density = 20%.

The parameter *K* determines the number of similar neighbors. To study the impact of *K* on the hybrid model HL-RW, we set the *K* increasing from 1 to 10. The other parameter *λ* is set to the default value, and the results are shown in the following Figure 7.

From Figure 7, it can be seen that in both response time and throughput datasets, the values of prediction error (Normalized Mean Absolute Error) undergo stable change, and are in a quite limited range. The optimal value can be achieved at *K* = 4, 5 or 6. On the one hand, it indicates that the proposed method is not sensitive to the setting of *K*, that is, the number of similar neighbors, to different QoS properties. This is important for improving the applicability of the method, since the method can be used in different QoS properties with one unified parameter setting. On the other hand, it can be inferred that a small number of similar neighbors are enough to guarantee the prediction accuracy, which is also beneficial to save the computation overhead.

### 6.6. Computation Overhead Comparison

In this section, we report the computation overhead of our proposed methods (UL-RW, SL-RW and HL-RW) and the compared well-known methods, in the response time dataset over all cases of training set densities. The parameters in all methods are set to the default values. The running time results are given in the following Table 5.

From Table 5, it first can be seen that service-based methods (IPCC, SL-RW) consume higher running time than user-based methods (UPCC, UL-RW). The reason is that in the dataset, the number of users (339) is much smaller than the number of services (5825), in an order of magnitude. Note that, in user-based methods and service-based methods, a necessary step is to compute the similarity between two users or two services, so the computation overhead of the two kinds of methods will be enlarged. Thus, the running time of user-based methods is clearly smaller than that of service-based methods.

Second, compared to traditional user-based methods (e.g., UPCC), the running time of our proposed user-based method UL-RW is only marginally higher than that of UPCC. Similarly, the difference between the running time of our proposed service-based method SL-RW and IPCC is also limited. As for the comprehensive methods, including WSRec, LACF SVD, the proposed method HL-RW also achieve competitively running time. In some cases, for example, in the training density being 10% to 20%, the running time of HL-RW is less than that of SVD.

In the experimental results of prediction accuracy comparison, from Table 3 and Table 4, we have seen that our proposed three methods all achieve higher results. Based on the running time comparison in this section, we can further see that the running time of our methods is competitively and applicable in practice.

## 7. Discussion and Summary

### 7.1. Summary of the Motivation

In the above sections, we have given a complete explanation of our work, including the research background, the proposed framework and methods, the experimental results and analysis, and some other parts. To give a deeper understanding of our work, in this section, we give a summarized discussion on the research motivation of our work. The motivations of our work mainly come from three aspects, and all the aspects are closely related to the real requirement of service recommendation in cyber-physical systems (CPS).

The primary motivation is the key goal of this paper, that is, to promote the prediction accuracy of QoS values as a criterion to select suitable services for target users. This motivation comes from the observation that the number of services in CPS is increasing quickly. So we need to tackle the urgent task, recommending or selecting appropriate services from a large number of candidates. In this paper, we focus on proposing methods, which utilize the non-functional properties (i.e., QoS) as the selection criteria.

The second motivation is to solve the sparsity problem in service recommendation in CPS. The sparsity problem is led by the limited service invocation records that a user can have, compared to the large whole number of all potential services. In this paper, we propose an extended similarity computation method, based on Euclidean distance, to solve the sparsity problem.

The third motivation is to study the role of network location as context information in QoS prediction, to study whether and how network location can improve the prediction accuracy. We observe that in CPS, network location is a piece of representative context information, but has been not yet given enough attention in QoS prediction. In this paper, we propose an extended random walking model leveraging network location information, to find similar neighbors and filter fake neighbors.

### 7.2. Discussion on the Network Location

In this paper, we compute the similarity of two users or services, partly based on the inference of similar relation from the network locations of users or services. This inference is further based on the observation that services in the same autonomous system (AS) are likely to have a similar network environment and thus tend to have similar QoS.

We leverage IP addresses and WSDL addresses, which are two types of network context information, to infer and build potential network relations among users and services. However, in the same autonomous system, in some cases, different services may be in heterogeneous network environments, because of the potentially diverse access technologies. So we propose a filtering strategy to filter fake neighbors. In detail, we propose a similar neighbor selection algorithm, which utilizes both the network information and QoS records to filter fake neighbors with abnormal QoS values. Thus in the case that the two users in the same AS are not neighbors, our proposed neighbor selection algorithm has the capability of identifying such fake neighbors.

We conducted comprehensive experiments on two datasets over different data densities, and we can see that our models achieve superior results in all cases. The high prediction accuracy can also indicate the effectiveness of the utilization of network location information.

## 8. Conclusions and Future Work

In this paper, we propose three location-based random walk models for QoS prediction in CPS. We propose a novel similarity computation method. On one hand, we propose a new distance measure. On the other hand, we take the network location information into the random walk model to generate a better neighborhood. We also propose a hybrid model, which can utilize the results of both individual models. We conduct extensive experiments in two large-scale real-world datasets, and the results verify the effectiveness of our models.

In the future, we plan to investigate the performance of our models on more QoS properties, such as reputation and reliability. Also, we are going to study the impact of time factor in service invocation, and further investigate ways of incorporating the time factor into the existing models.

## Figures and Tables

**Table 1:** A toy example of the user-service invocation matrix.

	Service 1	Service 2	Service 3	Service 4	Servcie 5
User 1	?	?	1.74	?	?
User 2	1.28	?	?	?	3.14
User 3	?	?	?	0.89	?
User 4	3.21	?	?	?	1.35

**Table 2:** Statistics of data.

Attributes	Numbers
the number of users	339
the number of services	5828
the number of invocation records	1,974,675
the number of user countries	30
the number of service countries	73
average value of response time	0.81
average value of throughput	44.03

**Table 3:** Accuracy comparison (a smaller value means higher accuracy).

Model	Training Set Density (TD)—Response Time Dataset							
	TD = 5%		TD = 10%		TD = 15%		TD = 20%	
	MAE	NMAE	MAE	NMAE	MAE	NMAE	MAE	NMAE
UserMean	0.8818	1.0873	0.8794	1.0832	0.8788	1.0832	0.8785	1.0837
ItemMean	0.7223	0.8904	0.7082	0.8723	0.7014	0.8642	0.7002	0.8630
UPCC	0.7568	0.9332	0.7137	0.8802	0.6311	0.7779	0.5919	0.7298
IPCC	0.7184	0.8851	0.7345	0.9061	0.6991	0.8617	0.6503	0.8013
WSRec	0.6832	0.9409	0.6306	0.8390	0.6137	0.7810	0.6020	0.7545
LACF	0.6575	0.8476	0.6398	0.8011	0.6023	0.7425	0.5723	0.7055
SVD	0.5793	0.7142	0.5683	0.7006	0.5430	0.6704	0.5328	0.6568
**SL-RW**	**0.5885**	**0.6054**	**0.5036**	**0.5609**	**0.4763**	**0.5254**	**0.4598**	**0.5026**
**UL-RW**	**0.5289**	**0.6481**	**0.4667**	**0.5782**	**0.4490**	**0.5489**	**0.4302**	**0.5297**
**HL-RW**	**0.5172**	**0.6374**	**0.4604**	**0.5580**	**0.4316**	**0.5274**	**0.4128**	**0.5069**

**Table 4:** Accuracy comparison (a smaller value means higher accuracy).

Model	Training Set Density (TD)—Throughput Dataset							
	TD = 5%		TD = 10%		TD = 15%		TD = 20%	
	MAE	NMAE	MAE	NMAE	MAE	NMAE	MAE	NMAE
UserMean	51.032	1.1644	52.822	1.1665	51.051	1.1597	51.490	1.1584
ItemMean	32.386	0.7389	32.226	0.7117	31.889	0.7244	31.895	0.7175
UPCC	29.157	0.6653	25.464	0.5624	22.270	0.5059	20.479	0.4607
IPCC	47.748	1.0894	47.098	1.0401	40.802	0.9321	39.505	0.8887
WSRec	30.502	0.6783	26.532	0.5892	22.025	0.5048	20.213	0.4587
LACF	28.612	0.6543	25.451	0.5714	22.403	0.5123	20.105	0.4439
SVD	35.972	0.7072	32.563	0.6753	31.852	0.6528	29.774	0.6303
**SL-RW**	**29.449**	**0.6889**	**27.170**	**0.6098**	**24.237**	**0.5499**	**23.438**	**0.5314**
**UL-RW**	**23.279**	**0.5352**	**20.740**	**0.4669**	**19.123**	**0.4349**	**17.888**	**0.4071**
**HL-RW**	**23.263**	**0.5348**	**20.428**	**0.4511**	**18.934**	**0.4315**	**17.315**	**0.4028**

**Table 5:** Running time comparison (unit: second).

Model	Training Set Density (TD)—Response Time Dataset			
	TD = 5%	TD = 10%	TD = 15%	TD = 20%
	Running Time (s)	Running Time (s)	Running Time (s)	Running Time (s)
UPCC	22.139	43.727	77.589	136.889
IPCC	358.659	612.326	941.629	1243.632
WSRec	383.324	659.884	1012.265	1372.266
LACF	267.514	483.721	831.260	1152.265
SVD	399.347	811.453	1232.871	1982.789
**UL-RW**	**31.403**	**59.166**	**92.570**	**148.618**
**SL-RW**	**383.226**	**726.570**	**1053.269**	**1358.321**
**HL-RW**	**418.009**	**789.102**	**1151.890**	**1509.598**
